# Template‐based field map prediction for rapid whole brain B_0_ shimming

**DOI:** 10.1002/mrm.27020

**Published:** 2017-11-28

**Authors:** Yuhang Shi, S. Johanna Vannesjo, Karla L. Miller, Stuart Clare

**Affiliations:** ^1^ Wellcome Centre for Integrative Neuroimaging University of Oxford, John Radcliffe Hospital Oxford United Kingdom

**Keywords:** B_0_ shimming, shim calculation, brain imaging, field map database

## Abstract

**Purpose:**

In typical MRI protocols, time is spent acquiring a field map to calculate the shim settings for best image quality. We propose a fast template‐based field map prediction method that yields near‐optimal shims without measuring the field.

**Methods:**

The template‐based prediction method uses prior knowledge of the B_0_ distribution in the human brain, based on a large database of field maps acquired from different subjects, together with subject‐specific structural information from a quick localizer scan. The shimming performance of using the template‐based prediction is evaluated in comparison to a range of potential fast shimming methods.

**Results:**

Static B_0_ shimming based on predicted field maps performed almost as well as shimming based on individually measured field maps. In experimental evaluations at 7 T, the proposed approach yielded a residual field standard deviation in the brain of on average 59 Hz, compared with 50 Hz using measured field maps and 176 Hz using no subject‐specific shim.

**Conclusions:**

This work demonstrates that shimming based on predicted field maps is feasible. The field map prediction accuracy could potentially be further improved by generating the template from a subset of subjects, based on parameters such as head rotation and body mass index. Magn Reson Med 80:171–180, 2018. © 2017 The Authors Magnetic Resonance in Medicine published by Wiley Periodicals, Inc. on behalf of International Society for Magnetic Resonance in Medicine. This is an open access article under the terms of the Creative Commons Attribution License, which permits use, distribution and reproduction in any medium, provided the original work is properly cited.

## INTRODUCTION

Shimming is a technique for homogenizing the static magnetic field in MRI 1, 2, 3 and spectroscopy 4. Most MR techniques, especially echo‐planar imaging and steady‐state free precession, benefit from a relatively homogeneous magnetic field. For the human brain, the most prominent field heterogeneity occurs around the sinuses and ear cavities, because of the high magnetic susceptibility difference between air and tissue. The heterogeneity of the magnetic field in those regions leads to significant image artifacts, such as signal loss and image distortions. For ultrahigh‐field MRI, these artifacts are accentuated, as field inhomogeneity scales with background field strength, and shimming becomes a crucial limitation on image quality.

Most clinical MR scanners are equipped with room‐temperature shim coils that generate spherical harmonic magnetic fields, which counteract distortions of the main magnetic field caused by the presence of the subject. Most 3T systems are equipped with second‐order shim coils, whereas some ultrahigh‐field systems have even higher orders available. Typically, MRI studies will acquire a field map as one of the first scans. The field map is used to calculate a current for each shim coil that will result in a total field that is maximally homogeneous. A typical static B_0_ shimming procedure for a human head can take approximately 30 s; however, the time for shimming will be scaled if the field map needs multiple acquisitions to accurately characterize the field. This is particularly the case if the subject is poorly shimmed to start with.

For many applications, particularly in the clinical setting, cutting exam time is of great importance. One conventional B_0_ mapping technique is 3‐dimensional (3D) volume‐based gradient‐echo field mapping, which estimates the field based on phase images acquired at different echo times (TEs). It provides a robust estimation of the B_**0**_ field distribution, but often takes a minute or more to acquire. Multi‐echo field mapping can improve robustness further, but at the expense of increased scan time 5. Methods for accelerating the B_0_ field mapping have been investigated in the last decade. Single‐shot techniques, such as echo‐planar imaging (EPI) 6, 7 and spiral‐based 8, 9 acquisitions, have been used for field mapping. Although these methods are fast, distortion and signal loss in the acquired EPI or spiral images will be translated into the field maps and will affect the accuracy of shim determination. Alternatively, balanced steady‐state free‐precession sequences 10 have been demonstrated to provide B_0_ field maps with high signal‐to‐noise ratio on a short time scale. However, this performance is degraded if a wide range of frequencies is present in image voxels. In that case, the field estimation will be not accurate in regions of large field inhomogeneity (e.g., close to sinuses or ear cavities).

In addition to 3D volume‐based field mapping methods, projection‐based methods 11, 12, 13, 14 are used, which allow rapid shim determination based on the B_0_ field distribution information along the field map projections (e.g. the fast, automated, shimming technique by mapping along projections). This method is typically based on the assumption that the shim field can be well described by a sum of spherical harmonics, which is good for local shimming targets, such as spectroscopy. However, it is not applicable for some other shimming applications, such as slice‐wise dynamic shimming, because of limited spatial field distribution information available for determining shim settings for thin slices.

Instead of measuring the field directly, it has been suggested to computationally model the susceptibility‐induced field inhomogeneity 15, 16, 17, 18, 19. In these methods, the field inhomogeneity is calculated by convolving a 3D dipole field kernel with a predicted susceptibility distribution. However, this relies on the use of additional high‐resolution anatomical images acquired from both CT and MRI to build a subject‐specific accurate susceptibility model for B_0_ field estimation. The high computational time and memory cost make this method inconvenient for clinical applications.

In this work, we propose a rapid template‐based field map prediction to estimate field inhomogeneity across the brain in a short timeframe, which can reduce the time spent on the field map acquisition at the shimming stage. The prediction method uses prior knowledge of the B_0_ field distribution in the human brain based on a large database of field maps, together with the basic subject‐specific structural information from a localizer scan. In clinical MR studies, a localizer is typically acquired for slice positioning, so the proposed method adds no time to the routine MR procedure. The performance of static shimming using the predicted field map method is evaluated on 143 subjects in simulation and 7 subjects in experiment.

## METHODS

**Figure 1:**
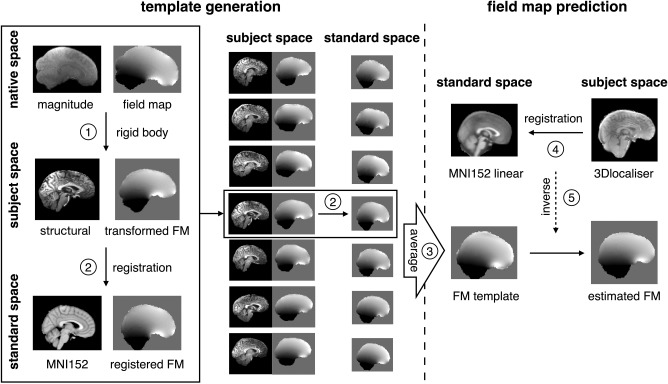
Procedures for template‐based field map prediction, which consists of two main stages: template generation and field map prediction. The field map template generation is conducted before the experiment. For each subject in the database, (1) the magnitude image of the field map acquisition and the field map are aligned to the T_1_‐weighted image and then (2) the aligned field map is warped to standard space by applying the registration transformation from the T_1_‐weighted image to the MNI (Montreal Neurological Institute) template. (3) The field map template is obtained by averaging all the warped field maps in standard space. The field map prediction is conducted on the day of scan. (4) When the quick localizer is acquired, it is registered to standard space. Following this, (5) the generated inverse registration is applied to the general field map template to get the estimated field map corresponding to the scanned subject.

The field map prediction method relies on determining a field map template, and using that to estimate a particular subject's field map. The method therefore consists of two main stages: field map template generation and subject‐specific field map prediction. The first stage is to determine a template of the characteristic susceptibility‐induced field distribution in the brain, based on measurements from a wide range of subjects. In the second stage, the field map for a specific subject is predicted based on the template together with a quick structural scan (e.g. localizer). The method is illustrated step by step in Figure 1.

The experiments were performed on a Siemens Magnetom whole‐body 7T MRI scanner (Siemens Healthineers, Erlangen, Germany) equipped with 70 mT/m gradients and second‐order spherical harmonic shim coils. Image data were acquired with a 32‐channel head coil (Nova Medical, Wilmington, MA, USA). A total of 176 healthy subjects (age range 20–66) were scanned as part of separate studies carried out by different researchers on the scanner. The scans were acquired in compliance with approved ethics protocols, which also allowed for anonymous data to be shared for the purpose of this study. In positioning the subjects in the scanner, no special instruction in terms of head position or rotation angle was given. For each subject, the vendor‐supplied routine shimming procedure was applied before any further scans. The shim settings were recorded in Digital Imaging and Communications in Medicine files, enabling later removal of the baseline field. All study protocols acquired a B_0_ field map for the purpose of removing image distortion in postprocessing, and a T_1_‐weighted structural scan. The field map was acquired with a 2‐dimensional dual gradient‐echo sequence (voxel size = 2 × 2 × 2 mm^3^, TE1 = 4.08 ms, ΔTE = 1.02 ms, flip angle = 39°, repetition time (TR) = 620 ms, slices = 64). The T_1_‐weighted structural image was acquired with a 3D inversion recovery gradient‐echo sequence (TR = 2200 ms, TE = 2.82 ms, inversion time = 1050 ms, voxel size = 1 × 1 × 1 mm^3^, GRAPPA (GeneRalized Autocalibrating Partial Parallel Acquisition) factor = 4). To ensure that all field maps had sufficient brain coverage, field map scans with less than 90% brain content coverage, as calculated by the ratio of brain contained in the field map to that in the T_1_‐weighted image, were rejected.

### Field Map Template Generation

Using the field map database described previously, a characteristic field‐distribution template used for field map prediction was generated as follows. First, for each brain in the database, the field maps were unwrapped using PRELUDE (Phase Region Expanding Labeller for Unwrapping Discrete Estimates) 20. The baseline shim field was removed using the recorded shim settings from the DICOM (Digital Imaging and Communications in Medicine) file and premeasured spatial field distributions of each individual shim coil. Specifically, the spatial distribution of magnetic field generated by each shim term was measured on an 18‐cm‐diameter spherical oil phantom using a 2‐mm isotropic field mapping sequence. A range of shim values (±500 mT/m for first‐order and ±3000 mT/m^2^ for second‐order shims, 20% increments) were measured and the data were decomposed into spherical harmonic functions from zeroth to fourth order using a least‐squares method. Second, each T_1_‐weighted structural image and the magnitude image from the field map acquisition were brain‐extracted using BET (FMRIB's Brain Extraction Tool) 21. Then the magnitude image from the field map data was aligned to the T_1_‐weighted image using the rigid‐body registration in FLIRT (FMRIB's Linear Image Registration Tool) 22, and the resulting transform was applied to the field map. Third, the T_1_‐weighted image was moved from subject space to standard space, as defined by the MNI (Montreal Neurological Institute) template 23, using FLIRT with 12 degrees of freedom. The corresponding field maps were warped to standard space by applying the registration transformation obtained from the T_1_‐weighted image. The reason for using the T_1_‐weighted structural image as an intermediate for the registration to standard space, instead of just the field map magnitude image, is that T_1_‐weighted images acquired in the experiments cover the entire brain, thereby providing more structural information for the registration. In the final step, the field map template was generated by averaging all of the registered field maps in standard space.

### Field Map Prediction

The subject‐specific field distribution is estimated based on the field map template and an acquired structural image. For an individual subject, the structural image was brain‐extracted and resampled down to 2‐mm isotropic, equivalent to the MNI standard images, to speed up computations of the further processing steps. The resampled structural was registered to standard space with FLIRT. Finally, the predicted field map was obtained by transforming the field map template to subject space using the inverse of the structural‐to‐standard space registration transformation.

### Shim Calculation

This method produces an estimate of the B_0_ field distribution in the individual subject, which can then be used for shimming. To calculate shim currents, measured field distributions from the first‐ and second‐order shims coils were fit to the predicted field map using a linear least‐squares approach 24. The goal was to minimize B_0_ inhomogeneity within the region defined by the brain extraction mask.

The feasibility of using the template‐based field map prediction for shimming was evaluated in comparison to a range of other potential shimming strategies in both simulation and experiment. To quantify the performance of the different approaches, the standard deviation of the residual field after shimming was calculated in each case.

### Simulation

The field map database contains a rich amount of information, so to evaluate what different features are contributing to shimming performance, a range of scenarios was simulated. For these simulations, we term this method the “average registered field map.” In the simulations, the brain structural information available in the T_1_‐weighted image was used to transform the template field map from standard space to the space of the subject of interest. The results were compared with a shim based on the actual field map for that subject (i.e., the “measured field map”).

We might expect that some brains in the database would act as a very good predictor of field and others much less so. To assess this, each individual field map in the database was transformed to the space of the subject of interest, and this field map was used to calculate shim terms. We term this the “registered field map.”

These registered field maps incorporate information about the brain geometry of the individual subjects. To evaluate whether this geometry information delivers better field map prediction for the shim settings calculation, an assessment without such information is useful. To do this we took the optimum shim for each individual subject in the database, without any transformation, and applied this to the subject of interest. We term this the “fixed shim.” Furthermore, we calculated the average of all of the fixed shim values in the database to yield a template shim setting, here termed the “average fixed shim.”

Then, to evaluate to what extent shimming quality will be improved using information from the database, a Monte Carlo simulation of random shims was implemented. The shims were sampled uniformly in the range between the minimum and the maximum of individual fixed shim values across all subjects in the database. We term this the “random shim.”

Finally, the default vendor‐calibrated shim for an oil phantom, the “tune‐up shim,” was used as a comparison to demonstrate the benefits of using field map information for shimming. Table 1 summarizes all of the comparisons.

**Table 1:** Terminology Used for Describing Different Shimming Methods

Term	Definition
Measured field map	Measured spatial field distribution of an individual subject
Individual registered field map	Field map generated by registering a measured field map of a particular subject to another subject
Averaged registered field map	Average of registered field maps for all of the subjects in the database
Individual fixed shim	Shims calculated based on the measured field map of an individual subject
Averaged fixed shim	The average of the individual fixed shims across all of the subjects in the database
Random shim	Monte Carlo simulation of shims sampled uniformly at random in the range between the minimum and the maximum of individual fixed shim values across all of the subjects in the database
Tune‐up shim	Calibration shim performed by manufacturer to optimize field homogeneity in an oil phantom

To quantify the performance of the different shimming methods, a leave‐one‐out cross‐validation analysis was applied to each subject in the database. For each cross‐validation step, 142 of 143 subjects were assigned into a training group used to generate a field map template. The remaining one subject was used to evaluate the shimming performance of each of the shimming methods in Table 1. The evaluation yielded one shim setting per subject for the averaged registered field map, the averaged fixed shim, the tune‐up shim, and the measured field map, whereas for the individual registered shim, individual fixed shim and random shim, a whole distribution was generated per subject. The full set of simulations was repeated for each subject in the database. All simulations were carried out in the MATLAB R2015a environment (MathWorks Inc, Natick, MA, USA).

### Experiment

To evaluate the performance of the proposed template‐based shimming in a realistic setting, experiments were conducted on seven volunteers who were not included in the database of field maps. In the experiments, a routine 3D localizer (field of view = 260 × 260 × 201.6 mm^3^, voxel size = 1.4 × 1.4 × 1.4 mm^3^, TE = 1.56 ms, TR = 3.6 ms, GRAPPA factor = 3, acquisition time = 19 s) was acquired to get the brain geometric information of the scanned subject, to generate the predicted field map. The template‐based shimming was compared with a number of different methods used in the simulations. For each method, shimming performance was quantitatively evaluated by the standard deviation in field maps (voxel size = 2 × 2 × 2 mm^3^, TE1 = 4.08 ms, ΔTE = 1.02ms, slices = 80, flip angle = 39°, TR = 640 ms, field of view = 220 × 220 mm^2^, acquisition time = 2:24 min) acquired with the respective shim settings, and qualitatively evaluated by signal loss and distortion artifacts in EPI images (voxel size = 2 × 2 × 2 mm^3^, slices = 80, TR = 4610 ms, TE = 25 ms, field of view = 220 × 220 mm^2^, GRAPPA factor = 2, echo spacing = 0.72 ms). A B_0_ field map and an EPI volume was acquired with each of the following shim settings:

1. Tune‐up shims;
2. Shims calculated by the routine vendor‐supplied shimming method. This measures the field distribution in the subjects’ brains with a dual‐echo steady‐state sequence, and calculates the shim settings designed to minimize field gradients over the brain. The procedure can be performed iteratively, updating the baseline shim settings to the newly calculated ones in each step, incurring an increase in the time required for shimming. Here, we used shim settings resulting from a single iteration and from three iterations, for comparison;
3. The averaged fixed shim from the database;
4. The proposed template‐based shimming (i.e., shims calculated based on the averaged registered field map, using the 3D localizer to transform the map to subject space); and
5. Shims calculated based on a measured B_0_ field map (parameters as defined previously).

### Third‐Order Shimming

All evaluations so far were performed with up to second‐order spherical harmonic shimming. However, some MR systems have up to third‐order shim hardware, which is not available on our system. To assess whether the field map template approach provides meaningful spatial field information for third‐order shimming, additional simulations were performed on the data from the seven subjects not included in the database. The simulations compared second‐ and third‐order shimming based on the measured field map and the averaged registered field map.

## RESULTS

**Figure 2:**
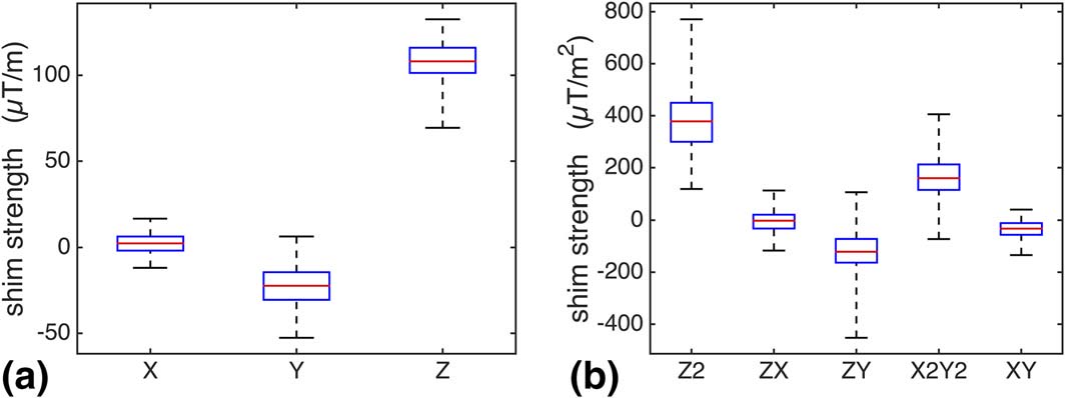
First‐order (**a**) and second‐order (**b**) shims calculated based on measured field maps for all 143 subjects in the database. For the box plot of each shim term, the five line markers from bottom to top represent the minimum, 25th percentiles, mean, 75th percentiles, and maximum of the shim value distribution. The x‐, y‐, and z‐axis corresponds to the left–right, anterior–posterior, and superior–inferior directions of the subject coordinates, respectively.

Figures 2a and 2b show the range and average of individual shims settings of the first‐order and second‐order terms, respectively, calculated based on the measured field map, for all 143 subjects in the database. It is evident that the most prominent shim offset is on shims having strong spatial field dependence in the inferior–superior direction (z‐axis), especially the Z and Z2 shims. The second dominant shim offset is in the anterior–posterior direction (y‐axis), whereas the least is along the left–right direction (x‐axis). The first‐order shims show relatively high consistency among different subjects, whereas second‐order shims vary more relative to the mean across subjects.

**Figure 3:**
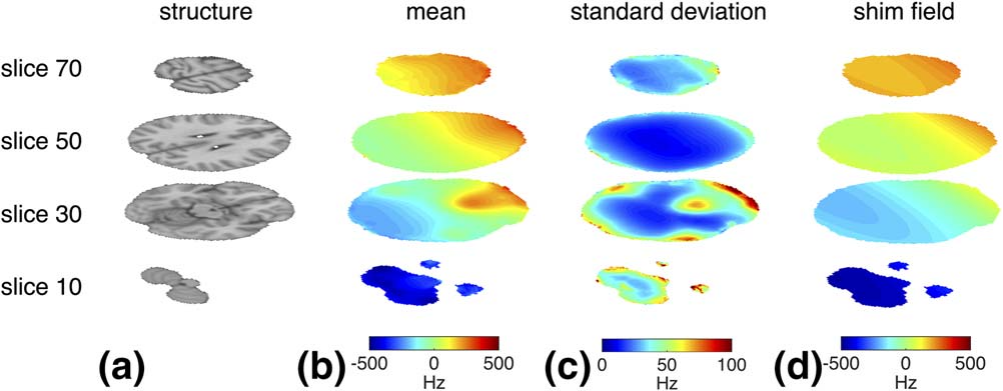
Example slices of the MNI brain structural template (**a**), mean (**b**) and standard deviation (**c**) of all 143 registered field maps, and the field compensated for by the shim field (**d**) generated by the averaged fixed shim.

Figures 3b and 3c present the mean and standard deviation, respectively, of the field distribution from all 143 registered field maps in standard space, together with the field compensated for by the averaged fixed shim (Fig. 3d). The mean field distribution indicates the common field features across different subjects in the database. This is dominated by a gradient in the inferior–superior and the anterior–posterior direction, on top of which there is a more localized field deviation in the region of the orbitofrontal lobe. The standard deviation represents the variation in field distribution among different subjects. The largest intersubject field variations are in regions near the frontal sinuses and the ear cavities. The field distribution in these regions has higher‐order spatial definition than can be generated by second‐order shim coils for whole‐brain shimming. Thus, the mean shim compensation field largely represents the long‐ranging field gradients, as is evident from Figure 3d.

**Figure 4:**
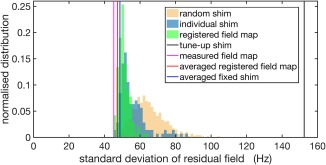
Comparison of simulated shimming performance using different shimming methods indicated in Table 1 for an example subject in the database. The x‐axis shows the standard deviation of residuals after shimming. The y‐axis indicates normalized distribution of the number of samples producing certain shimming results. The vertical reference lines from left to right indicate the standard deviation of residuals after shimming with the measured field map of the selected subject (pink line), the averaged registered field map (red line), the averaged fixed shim (blue line), and the tune‐up shim (black line). The histograms indicate the shimming results of using individual registered field maps from the other subjects in the database (blue histogram), individual fixed shims (red histogram), and random shims (orange histogram).

Figure 4 compares the residual B_0_ standard deviation following simulated shimming using the shim calculation methods listed in Table 1, for an example subject in the database. The pink, red, blue, and black lines indicate the residual B_0_ standard deviation using the measured field map, the averaged registered field map, the averaged fixed shim, and the tune‐up shim, respectively. Using the averaged registered field map, the B_0_ standard deviation within the brain region of interest is reduced from 152 Hz (tune‐up shim) to 47 Hz, only 2 Hz worse than the measured field map method. The averaged fixed shim performed almost as well in this subject, yielding 49 Hz residual B_0_ standard deviation.

The green, blue, and orange histograms (normalized to unit area) show the residual B_**0**_ standard deviation using individual registered field maps, individual fixed shims and random shims, respectively. Even the random shims yielded a distribution of residual standard deviation shifted toward much lower values than the tune‐up shim, which is unintuitive at first glance, but is likely caused by the range of shim settings being limited to the maximum and minimum values observed in the database. Using the individual fixed shims shifted the mode of the distribution further toward lower residual standard deviation, likely because these represent a “valid” combination of shim terms corresponding to a real subject. Finally, the individual registered field maps yielded a narrower distribution and reduced the tail of poorly performing shims. Interestingly, the averaged fixed shim and the averaged registered field map both yielded lower residual standard deviation than the mode of the corresponding distributions.

**Figure 5:**
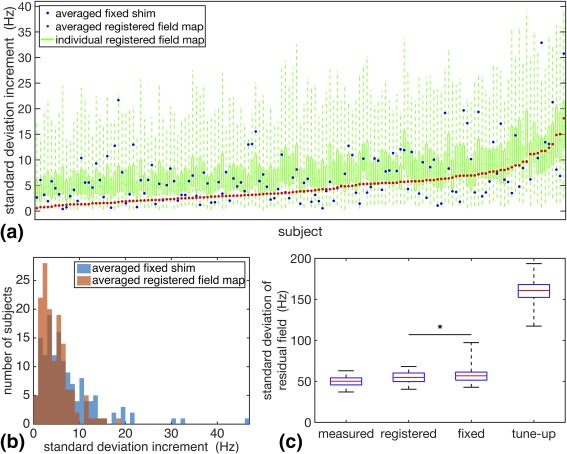
Comparison of the simulated shimming performance of the different shimming methods across all subjects in the database. **a**: Relative increase in standard deviation of the residual field with the averaged registered field map (red dots), the averaged fixed shim (blue dots), and the individual registered field maps (green box plots), as compared with using the measured field map. The subjects are sorted according to the values of the relative standard deviation of the averaged registered field map. **b**: Distribution of relative increase in standard deviation of the residual field using the averaged registered field map (red histogram) and the averaged fixed shim (blue histogram), as compared with using the measured field map. **c**: Distribution of standard deviation of the residual field using the measured field map, the averaged registered field map, the averaged fixed shim, and the tune‐up shim for all subjects in the database. The nonparametric Wilcoxon signed‐rank test resulted in *P* < 0.001.

The results from all 143 trials from the leave‐one‐out simulation are depicted in Figure 5. In 60% of the cases (86 subjects), the averaged registered field map provided standard deviation of residuals no more than 5 Hz higher than those based on a measured field map. Poor performance of the averaged registered field map generally correlated with a larger spread and higher average of the residual standard deviation of the individual registered field maps. This result has the intuitive interpretation that if the registered field maps used to generate the field map template are not representative of the scanned subject, the averaged registered field map cannot provide good shimming results. However, for all subjects in the database there existed a subset of individual registered field maps that provided a good prediction of the field. In 64% of the cases (91 subjects), the averaged registered field map provided lower standard deviation than the averaged fixed shim. Comparing the distribution of the residual standard deviation shows that the averaged registered field map reduced the tail of poorly performing shim settings as compared with the averaged fixed shim (Fig. 5b).

As illustrated in Figure 5c, the mean standard deviation of residuals across all subjects in the database was 50 Hz using the measured field map, compared with 160 Hz for the tune‐up shim. Using the averaged registered field map for shimming brings the standard deviation of residuals down to, on average, 55 Hz. The average fixed shim was also effective in homogenizing the magnetic field over the brain, yielding a mean standard deviation of 57 Hz. To evaluate whether the averaged registered field map statistically outperforms the fixed shim, a nonparametric Wilcoxon signed‐rank test was carried out. This demonstrated that there is a significant (*P* < 0.001) improvement in shimming performance using the averaged registered field map, as compared with the averaged fixed shim.

**Figure 6:**
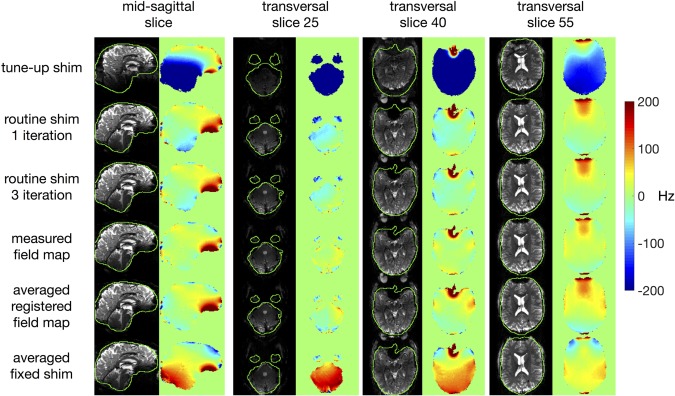
Field maps and echo‐planar images acquired using different shimming methods in one subject (subject C) not included in the database. The columns from left to right indicate midsagittal images (**a**) and transversal images at slice number 25, 40, and 55 (**b**–**d**). The rows (from top to bottom) indicate the shimming results using (i) the tune‐up shim, (ii) the vendor‐supplied routine shim with one iteration, (iii) the vendor‐supplied routine shim with three iterations, (iv) the measured field map, (v) the averaged registered field map (i.e., the template‐based field map prediction), and (vi) the averaged fixed shim.

Figure 6 shows sagittal and transversal views of B_0_ field maps and EPI images acquired on one of the subjects (subject C) using different shimming strategies. To visualize the magnitude of the EPI distortion artifacts, brain outlines based on the magnitude images of the field map acquisition are overlaid. In the EPI images, substantial gain of signal and reduction of distortion is visible for all shimming methods compared with the tune‐up shim. In the B_0_ field maps, the least improvement is observed in regions where air meets tissue (sinuses and ear cavities), as these cannot be fully compensated with static second‐order shimming only. For most regions of the brain, the shimming performance of using the averaged registered field map is comparable to the vendor routine shim and the shimming based on the measured field map. However, somewhat lower field homogeneity is observed in the frontal and temporal lobes, using the averaged registered field map. As the largest intersubject field variation is located in these regions (Fig. 3c), a general field template is always unlikely to perform equally well there. In this example case, the averaged fixed shim provided a more homogeneous field than the other methods in the frontal lobes. However, this came at the cost of overall lower field homogeneity in other regions and was not consistent across all subjects.

**Figure 7:**
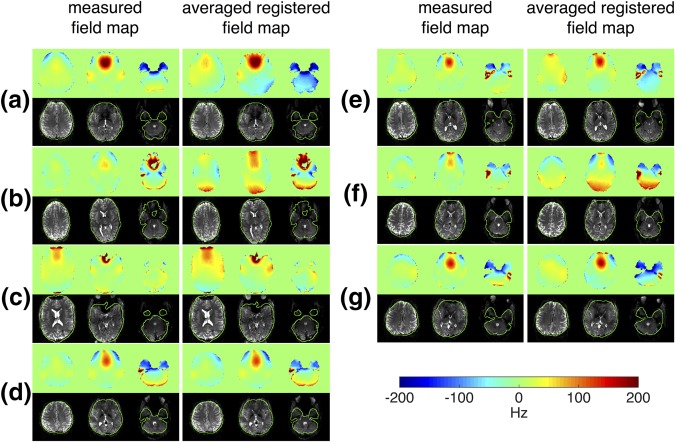
Experimentally acquired field maps and echo‐planar images shimmed using the measured field map and the averaged registered field map for seven subjects (A–G) not included in the database.

Figure 7 shows the experimentally measured B_0_ field maps and EPI images with shimming based on the averaged registered field map and the measured field map for all seven subjects (A–G). In five of the subjects (A, C, D, E, and G), the averaged registered field map produced results that were very comparable to using a measured field map. For subjects B and F, poor field inhomogeneity was seen at the back of the brain when using the template method. This is most likely because the subjects were scanned with large head rotation angles, which makes the template‐based prediction less reliable.

**Figure 8:**
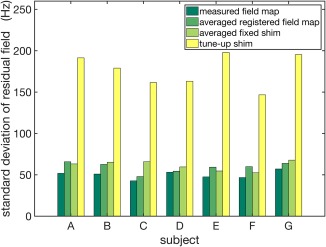
Standard deviation of the residual field within the brain after shimming using the measured field map, the averaged registered field map, the averaged fixed shim, and the tune‐up shim for seven subjects (A–G) not included in the database.

Quantified shimming results of the B_0_ field homogeneity using the different shimming methods are indicated in Figure 8. For all subjects (A–G), both the averaged registered field map and the averaged fixed shim delivered lower standard deviation of the residual field than the tune‐up shim. Specifically, static shimming using the averaged registered field map reduced the standard deviation from 176 to 59 Hz on average, which deviated from results using the measured field maps by 9 Hz. The averaged fixed shim provided a residual standard deviation of 61 Hz on average, comparable to the averaged registered field map.

**Figure 9:**
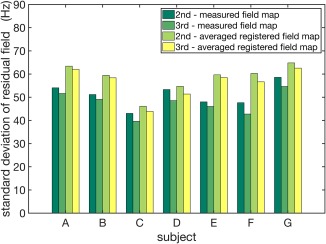
Standard deviation of the residual field within the brain after second‐ and third‐order simulated static shimming using the measured field map and the averaged registered field map for seven subjects (A–G) not included in the database.

These results suggest that an averaged registered field map offers useful information on the field distribution of the brain for second‐order static shimming. The residual standard deviation after simulated second‐ and third‐order shimming based on the averaged registered field map and the measured field map is shown in Figure 9. Third‐order shimming leads to a further reduction of the field inhomogeneity by 3.4 Hz on average when based on the measured field map, and by 2.1 Hz when based on the averaged registered field map.

## DISCUSSION

We have presented a rapid template‐based field map prediction approach capable of providing a quick estimation of the field distribution of scanned subjects using only a field map database and a localizer scan. The field distribution in an individual subject's brain is determined by the susceptibility distribution in the head and body, and the positioning angle relative to the main magnetic field. A simple rigid‐body transformation of a template B_0_ field map would therefore not be expected to provide a highly accurate predictor of the field. However, the results shown here suggest that such an approach may be feasible for the purpose of whole‐brain shimming. Both simulations and experiments demonstrate that shimming based on the field map template can substantially reduce the large‐scale field inhomogeneity over the brain. In practice, if the shim quality provided by the prediction method is insufficient in individual cases, it still provides a good initial estimate of the shim. The method would therefore reduce the need for multiple iterations in the shim determination.

The reasons behind the feasibility of using a general field map template for static shimming are two‐fold. First, in human populations, there is a high degree of similarity of the field distribution in the brain among different subjects. Averaging the registered field maps smoothes out individual differences, while maintaining the dominant field distribution. In addition, second‐ or third‐order spherical harmonic shim fields are relatively lower in spatial order compared with the field across the brain. As demonstrated here, the largest intersubject field variations are in areas of localized field that cannot be shimmed with low‐order spherical harmonic shimming. Conversely, the low‐order field component that can be addressed by spherical harmonic shimming is fairly consistent among subjects.

The dominant field inhomogeneity over the brain is a gradient along the z‐direction, which is associated with the field induced by the body, especially structures above the shoulders (e.g., the jaw and neck) 15. Because of the high similarity of the low‐order fields across subjects, especially along the z‐axis, even just the range of individual shim settings over the database carries useful information for shimming. This was demonstrated by the Monte Carlo simulation of random shims, which even in the worst cases reduced field inhomogeneity within the brain by almost a factor of two compared with the tune‐up shims, which are calculated from a phantom.

Surprisingly good shim performance was also achieved with the fixed shim approach, using no subject‐specific information. The mean residual field inhomogeneity of the fixed shim was almost as low as that of the averaged registered field map, and it even provided better shim quality in individual cases. Therefore, both methods appear to be capable of delivering good whole‐brain shimming in most subjects, within a very short timeframe. The fixed shim has the advantage that it is as quick and straightforward to implement as standard shim setting on any system. The performance of the fixed shim, however, was somewhat less robust than for the prediction method. In simulation, two of three subjects showed better shim performance with the averaged registered field map, and the variance of the residual field inhomogeneity was lower. Despite the small difference in average residual field inhomogeneity between the two approaches, it was statistically highly significant.

The main difference between the two methods is that the averaged registered field map incorporates information about the subject's head size and position. Because the center of the shim field is fixed in the scanner, the fixed shim is sensitive to differences in head positioning. In our data, the fixed shim approach was probably helped by the fact that the very tight head coil on our 7T scanner leaves little room for differences in head positioning. The provision of a very clear land marking cross on the head coil also reduces variability in positioning. The information about the subject's geometry in the prediction method comes from a quick localizer scan, which for most MR applications would be acquired anyway. The prediction method therefore yields a small, but significant, gain, at virtually no extra cost in time. In our current “offline” implementation, the total time for field map prediction, along with shim determination, just takes a few seconds. This is about one order of magnitude faster than the vendor routine shimming method, which takes approximately 30 s per iteration. Furthermore, graphics processing unit–based parallel computing techniques, and more integrated workflows with the scanner software, would help to accelerate the field map prediction and shim calculation processes even further 25.

The prediction method relies on a standard brain atlas as reference, to which different subjects’ structural images and field maps are registered for template generation. The standard brain atlas, however, is not representative for all population groups. For example, as the brain size changes as a function of age 26, subjects of different age ranges may not be well represented by the standard atlas. The size of the subjects’ chest cavities will also have an effect on the field distribution in the head. Standard templates stratified by age or weight could be investigated to improve the registration for specific target population groups. For subjects who have obvious anatomical changes as a result of neuropathology or implants, registration to a standard template is unlikely to be sufficient.

The performance of the template‐based prediction is degraded if the scanned subject is not well represented by subjects in the database. As shown in Figure 3, as the overall shimming performance provided by the individual field maps in the database becomes more variable, the quality of shimming provided by the average template becomes worse. However, there are always some registered field maps that deliver satisfactory shimming results. If these subjects can be identified based on structural information, such as using the parameters of the linear registration matrix, it may be possible to improve the field map template by averaging the field maps of a subset of selected individuals that represent a good match. Head rotation angles may provide particularly relevant information for subject selection. Head positioning 27, in particular tilting 28, changes the susceptibility‐induced B_0_ field, which can lead to shim changes of more than 5% 29. If a large database of field maps is available, it should be possible to find subjects of similar brain shape and positioning, thereby further improving the reliability of the technique. Incorporation of advanced machine learning approaches, like the random forest and the neural network method, could potentially facilitate the selection of matching subjects.

The proposed template‐based field map prediction was implemented here for ultrahigh‐field brain imaging, using second‐order shimming. Simulations indicate that systems equipped with third‐order shim hardware could also benefit from the averaged registered field map. At higher field strengths, shimming becomes increasingly important. However, the prediction method in itself should be equally applicable to imaging at lower field strengths, which is more clinically relevant. Future work can be carried out to evaluate the performance of the prediction method on standard clinical 3T systems.

In conclusion, the template‐based field map prediction technique joins satisfactory shimming performance with speed in shim determination. A wide range of MR applications could therefore potentially benefit from using this method for shimming of the human brain.
